# The HDAC6 Inhibitor Trichostatin A Acetylates Microtubules and Protects Axons From Excitotoxin-Induced Degeneration in a Compartmented Culture Model

**DOI:** 10.3389/fnins.2018.00872

**Published:** 2018-11-29

**Authors:** Kelsey Hanson, Nan Tian, James C. Vickers, Anna E. King

**Affiliations:** Wicking Dementia Research and Education Centre, College of Health, University of Tasmania, Hobart, TAS, Australia

**Keywords:** microtubule acetylation, trichostatin A, axon degeneration, HDAC6, excitotoxicity

## Abstract

Axon degeneration has been implicated as a pathological process in several neurodegenerative diseases and acquired forms of neural injury. We have previously shown that stabilizing microtubules can protect axons against excitotoxin-induced fragmentation, however, the alterations of microtubules following excitotoxicity that results in axon degeneration are currently unknown. Hence, this study investigated whether excitotoxicity affects the post-translational modifications of microtubules and microtubule-associated proteins, and whether reversing these changes has the potential to rescue axons from degeneration. To investigate microtubule alterations, primary mouse cortical neurons at 10 days *in vitro* were treated with 10 or 25 μM kainic acid to induce excitotoxicity and axon degeneration. Post-translational modifications of microtubules and associated proteins were examined at 6 h following kainic acid exposure, relative to axon degeneration. While there were no changes to tyrosinated tubulin or MAP1B, acetylated tubulin was significantly (*p* < 0.05) decreased by 40% at 6 h post-treatment. To determine whether increasing microtubule acetylation prior to kainic acid exposure could prevent axon fragmentation, we investigated the effect of reducing microtubule deacetylation with the HDAC6 inhibitor, trichostatin A. We found that trichostatin A prevented kainic acid-induced microtubule deacetylation and significantly (*p* < 0.05) protected axons from fragmentation. These data suggest that microtubule acetylation is a potential target for axonal protection where excitotoxicity may play a role in neuronal degeneration.

## Introduction

Axon degeneration is a pathological process that occurs in several neurodegenerative diseases, and acquired forms of brain injury, and can occur independently of cell death (Lingor et al., 2012). The best described mechanisms of axon degeneration are axonal pruning, which occurs during development and Wallerian degeneration, which occurs following axon severing (Coleman, 2005; Coleman and Freeman, 2017). In contrast, the cellular process underlying axon degeneration in a range of neurodegenerative diseases and following brain injury are less well understood and are unlikely to be linked to direct axonal severing, but rather to a gradual series of biochemical changes following specific disease-associated pathological insults.

One such pathological insult, excitotoxicity, is a result of excessive stimulation by the excitatory neurotransmitter glutamate, leading to, for example, increased intracellular calcium and a cascade of harmful calcium-dependent events (Dong et al., 2009). Excitotoxic mechanisms have been implicated in Alzheimer's disease, traumatic brain injury, stroke, and amyotrophic lateral sclerosis, which all undergo some form of axon degeneration (as reviewed by Gibson and Bromberg, 2012; Lai et al., 2014; Siedler et al., 2014; Lewerenz and Maher, 2015). Our previous studies in compartmentalized cultured neurons have shown that excitotoxicity directed to the neuronal soma results in fragmentation of the unexposed axon (Hosie et al., 2012). This was significantly attenuated by treatment of the axons with the microtubule stabilizing drug, taxol, implicating microtubule disruption in the breakdown of the axon (King et al., 2013).

Microtubules are highly dynamic polymers of alpha and beta tubulin. Their ability to undergo rapid changes in polymerization state (termed dynamic instability) is essential for their function and is controlled by the binding of associate proteins (MAPs) as well as by their post-translational modifications (PTMs) (Horio and Murata, 2014). The microtubule disruption that occurs following excitotoxin exposure and leads to axon degeneration could result from depolymerization, potentially relating to changes to PTM or from altered MAP binding. However, microtubule PTMs and MAP binding are not independent, and some MAP binding capacity to microtubules can be influenced by microtubule PTMs (Howes et al., 2014). PTMs of microtubules include glutamylation, tyrosination, and acetylation. Acetylation, glutamylation and detyrosination are associated with stable microtubules, whereas tyrosination is associated with unstable and more dynamic areas of microtubules (Uchida and Shumyatsky, 2015). Thus, microtubule PTMs may be a potential target for therapeutic intervention. In this regard, drugs targeting acetylation such as HDAC6 (histone deacetylase 6) inhibitors have been investigated for the treatment of depression, cancer, stroke, environmental stress and Huntington's disease (Dompierre et al., 2007; Lazo-Gómez et al., 2013; Simoes-Pires et al., 2013; Jochems et al., 2014; Brindisi et al., 2016, 2018; Ceccacci and Minucci, 2016; Wang et al., 2016; Rao et al., 2017).

In this study we have investigated the temporal changes to microtubule PTMs and MAPs following an excitotoxic insult with a view to modifying microtubule stability to prevent axon degeneration. Furthermore, we also investigated the effect of the HDAC6 inhibitor, trichostatin A, on axon degeneration.

## Materials and methods

### Ethics and mice

All experiments and procedures were approved by the University of Tasmania Animal Ethics Committee (A12780 and A15121) and were in accordance with the Australian Guidelines for the Care and Use of Animals for Scientific Purposes. C57Bl/6 mice were housed in optiMICE cages on a 12-h light/dark cycle with free access to food and water.

### Primary cortical cell culture

Mouse cortical neuron cultures were prepared as previously described (King et al., 2013). Cortical neurons were prepared from embryonic day 15.5 (E15.5) C57Bl/6 mice. Females were sacrificed using CO_2_ and embryos were removed and decapitated. Heads were kept on ice during dissection to prevent tissue degradation. Cortices were removed using a dissecting microscope (Leica), dissociated from the meningeal layers and transferred to 5 ml Hanks Balanced Salt Solution (HBSS, Gibco). Trypsin (0.0125%) was added for 4 min at 37°C, followed by removal of HBSS and replaced with 1 ml of initial plating media [Neurobasal media (Gibco) containing 2% B27 supplement, 0.5 mM glutamine, 25 μM glutamate, 10% fetal calf serum and 1% antibiotic/antimiotic] and mechanically triturated with a 1 ml pipette. Cell density was assessed using a trypan blue exclusion assay and 200,000 cells were plated either into the soma compartment of microfluidic chambers (450 μm long, 10 μm wide, and 3 μm high barrier grooves, Xona Microfluidics), which allows fluidic isolation of cells from the axon (Taylor et al., 2006), or directly into the wells of poly-L-lysine coated culture plates. Cells were allowed to adhere to the coverslip (Livingstone) at 37°C for 30 min prior to adding initial plating media to the chambers and were incubated at 37°C overnight before initial media was replacement with subsequent plating media with no fetal calf serum and glutamate. Cells were grown to 10 days *in vitro* (DIV) at 37°C in 5% CO_2_.

### Pharmacological manipulation

Cells were treated with 0, 10, or 25 μM of kainic acid (Sigma, lot #SLBD1491V) in DMSO (Sigma, Lot # RNBF1056) at 10 DIV, a timepoint which corresponds to dense axonal growth in the axon compartment of microfluidic chambers (Hosie et al., 2012; Millet and Gillette, 2012). Cells in 12-well plates or the soma compartment of microfluidic chambers were exposed to kainic acid for 1, 6, and 18 h. For a set of experiments cells were treated with trichostatin A, an inhibitor of the deacetylating enzyme HDAC6, which promotes microtubule acetylation. Cells at 10 DIV in 12-well plates or axonal compartment of microfluidic chambers were treated with 10 or 100 nM trichostatin A (Sigma, Lot # 026M4036V) for 2 h before 6 h of kainic acid exposure prior to ELISA analysis for acetylated tubulin. The health of cells following TSA treatment was not significantly different from vehicle control, as determined using an alamarBlue assay (Supplementary Figure 1). For all pharmacological manipulations vehicle controls were performed and are reported. No significant difference was found in any measure for vehicles to untreated cells.

### Live cell imaging and quantitation of axonal degeneration

Microfluidic cultures were imaged on a Nikon TiE live cell microscope, with chambers maintained at 37°C. Imaging was performed prior to treatment and 18 h following treatment. Axonal side of the microfluidic chambers were imaged using 40 × objective lens to measure axon fragmentation and degeneration. A quantitative measure of axonal degeneration was obtained from five random regions of interest (200 × 200 μm) in the axonal chamber, at least 40 μm away from the microgrooves. Identical regions were imaged before and after treatment. Axon degeneration was calculated by counting degenerated axons. A degeneration index (DI) was determined by the following equation.

(1) $$\begin{array}{r} DI=\left(\frac{\left(\frac{axons\;degenerated\;before}{axons\;degenerated\;after}\right)}{total\;number\;of\;axons}\right)\times \;100 \end{array}$$

The percentage fragmentation refers to the percentage of axons the have fragmented after treatment, compared to the total number of axons before treatment. In our experiments we also analyzed 35, 50, and 100 μM of kainic acid in the cell culture using live imaging, however the axons were too degenerated to be able to target therapeutically.

### ELISA analysis of microtubule post-translational modifications

Plating media was removed from cells plated in 12-well trays, and cultures were rinsed with HBSS. Cells were harvested with RIPA buffer with protease (Complete^TM^ Mini Protease Inhibitor Cocktail tablets, Roche) and phosphatase inhibitors (Phosphatase Inhibitor Cocktail, A.G. Scientific). Samples were centrifuged at 13,000 rpm for 1 min and pellets were discarded. Samples were diluted at 1:300 in 50 μl bicarbonate/carbonate coating buffer (AbCam), added to 96-well plate and incubated overnight at 4°C. For the standard curve, protein samples were serially diluted at 1:100, 1:200, 1:400, and 1:800. A blank and no primary control were included to correct for ELISA results. Plate was washed with washing buffer (0.01M PBS with 0.05% tween-20) prior to blocking with blocking buffer (0.01M PBS with 5% fetal bovine serum) and incubated at 37°C for 30 min. Plates were washed prior to incubation with detecting antibodies (acetylated tubulin 1:500 mouse, Sigma; tyrosinated tubulin 1:500 rabbit, Millipore; tau 1:1,000 rabbit, DAKO; CRMP2 1:1,000 rabbit, Sigma; MAP1B 1:500 mouse, AbCam) diluted in blocking buffer for 1 h at room temperature. Plates were washed and incubated with species-specific HRP secondary antibody (DAKO) diluted in blocking buffer at 1:2,000 and incubated at room temperature for 45 min. Plates were washed and incubated with room temperature tetramethylbenzidine (TMB) substrate (Thermo Scientific, Lot # SA2328991) for 15 min. The reaction was stopped 0.1M H_2_SO_4_. Plate was read using plate reader at 450 nm.

### Western blot analysis of microtubule post-translational modifications

Plating media was removed from microfluidic chambers and culture was rinsed with HBSS. Axons were harvested with RIPA buffer with protease (Complete^TM^ Mini Protease Inhibitor Cocktail tablets, Roche) and phosphatase inhibitors (Phosphatase Inhibitor Cocktail, A.G. Scientific), with protein pooled from 2 to 4 chambers per treatment group. Denatured protein samples (20 μg) were electrophoresed into Bolt^®;^ 4–12% Bis-Tris gels (Invitrogen), transferred to PVDF membrane (Bio-Rad) and incubated overnight with primary antibodies acetylated tubulin (1:1,000 mouse, Sigma), tyrosinated tubulin (1:1,000 rabbit, Millipore), MAP1B (1:1,000 mouse, AbCam). The corresponding anti-rabbit or anti-mouse horseradish peroxidase conjugated secondary antibody (1:7,000, DAKO) was used as previously described (King et al., 2018). GAPDH (1:5,000 mouse, Millipore) was used as a loading control. Bands were visualized with enhanced chemiluminescence (ECL) solution-Luminata Forte Western horseradish peroxidase (HRP) substrate (Millipore) and images acquired with a Chemi-Smart 5000 Imaging System (Vilber Lourmat) equipped with Chemi-Capt 5000 software. Band intensity was measured as the integrated intensity using Fiji software. After standardizing to GAPDH, each value was calculated as a percentage of control samples (See Supplementary Figure 1).

### Statistical analysis

Values were reported as means ± standard error of the mean (SEM), with differences considered significant at *p* < 0.05. Differences for ELISA and axon degeneration counts were evaluated using one-way ANOVA, with Tukey's *post-hoc* test for multiple comparisons between groups. For kainic acid live imaging experiments two coverslips on five separate culture days were analyzed. For trichostatin A live imaging experiments, 1–2 coverslips from four separate culture days were analyzed. For whole cell kainic acid ELISAs, three coverslips per treatment from four separate culture days were used. For whole cell trichostatin A ELISAs, three coverslips per treatment from five separate culture days were used. For axon-only kainic acid ELISAs, cells were pooled from 2 to 3 chambers per treatment from four separate culture days. For axon-only trichostatin A ELISAs, cells were pooled from 2 to 3 chambers per treatment from five separate culture days. All statistical analysis and graphs were prepared in GraphPad Prism (v6.1).

## Results

### Model of excitotoxin-induced axon degeneration

**Figure 1:**
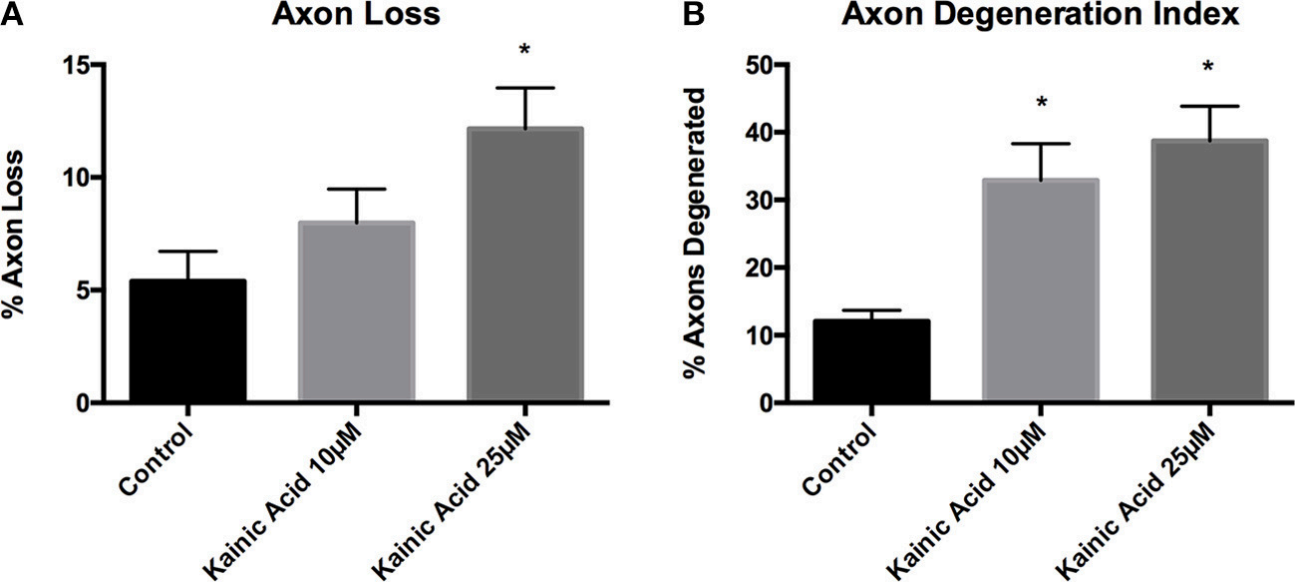
Axon degeneration and loss in microfluidic chambers after kainic acid treatment. Live imaging of the axonal compartment of microfluidic chambers were analyzed for **(A)** axon loss and **(B)** axon degeneration index after treatment with 10 or 25 μM kainic acid. Axon loss was significantly increased (*p* < 0.05) following 25 μM kainic acid treatment relative to control, whereas, 10 μM kainic acid did not cause significant axon loss compared to control. The axon degeneration index was significantly increased (*p* < 0.05) following either 10 or 25 μM doses of kainic acid compared to control. Bar graph represents mean ± SEM *p < 0.05 relative to control.

Microfluidic chambers allow compartmentalized separation of axons from the somatodendritic compartment, which permits treatment to be applied exclusive to either compartment of the chamber. To establish an acute model of excitotoxin-induced axon degeneration, mouse cortical neurons at 10 DIV were exposed to 10 and 25 μM kainic acid in the somatodendritic compartment for 18 h. Live imaging of axons was performed immediately prior to and post-treatment to determine a degeneration index. Axon loss was significantly increased (*p* < 0.05) at 25 μM kainic acid compared to control (Figure 1A). Axonal degeneration at both 10 and 25 μM kainic acid was significantly increased (*p* < 0.05) compared to control (Figure 1B).

### Microtubule alterations after excitotoxin-induced axon degeneration

**Figure 2:**
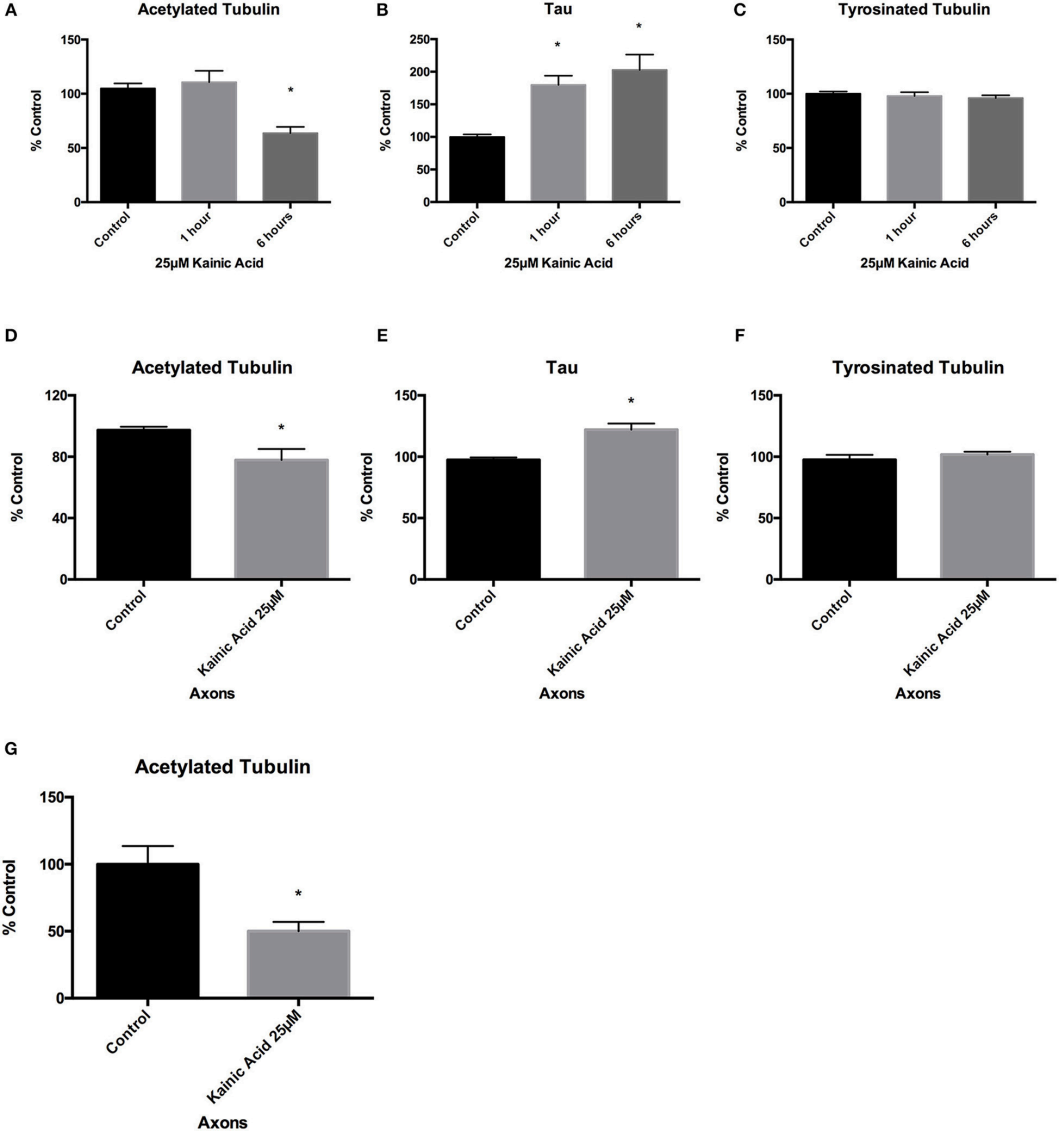
Changes to microtubules after kainic acid treatment. Changes to microtubule post-translational modifications and the microtubule-associated protein tau were investigated in both whole cultured neurons and isolated axons after treatment with 25 μM kainic acid. ELISA analysis of neurons at 1 and 6 h after 25 μM kainic acid treatment showed **(A)** a significant decrease (*p* < 0.05) in acetylated tubulin levels after 6 h and **(B)** a significant decrease (*p* < 0.05) in tau levels at both 1 and 6 h, relative to control. **(C)** Tyrosinated tubulin levels were unchanged compared to control. ELISA analysis of axons at 6 h post treatment with 25 μM kainic acid demonstrated **(D)** significantly decreased (*p* < 0.05) levels of acetylated tubulin, whereas **(E)** tau axonal levels were significantly increased (*p* < 0.05) at this timepoint relative to control. Axonal levels of **(F)** tyrosinated tubulin were unchanged compared to control. To confirm the effect of 25 μM kainic acid on acetylated tubulin in axons Western blot analysis was performed **(G)** demonstrating a significant decrease (*p* < 0.05). Bar graph represents mean ± SEM *p < 0.05 relative to control.

We next determined whether there were changes in microtubule PTMs and the microtubule-associated protein, tau, following 25 μM kainic acid exposure at 1 and 6 h, and whether these changes were related to downstream degeneration (Figure 2). Neuronal cells grown on 12-well trays and cells grown in the somatodendritic compartment of microfluidic chambers were exposed to 25 μM kainic acid for 6 h. ELISA analysis of neurons demonstrated that, at 1 h after 25 μM kainic acid treatment, acetylated and tyrosinated tubulin levels were unchanged, however the microtubule associated protein tau was significantly (*p* < 0.05) increased, compared to control (Figures 2A–C). At 6 h after 25 μM kainic acid treatment in both neurons and axons, acetylated tubulin levels were significantly (*p* < 0.05) decreased (Figure 2D). Tyrosinated tubulin levels were unchanged (Figure 2E), whereas, tau levels were also significantly (*p* < 0.05) increased (Figure 2F). Western blot analysis of acetylated tubulin levels in axons confirmed these were also significantly (*p* < 0.05) decreased (Figure 2G).

**Figure 3:**
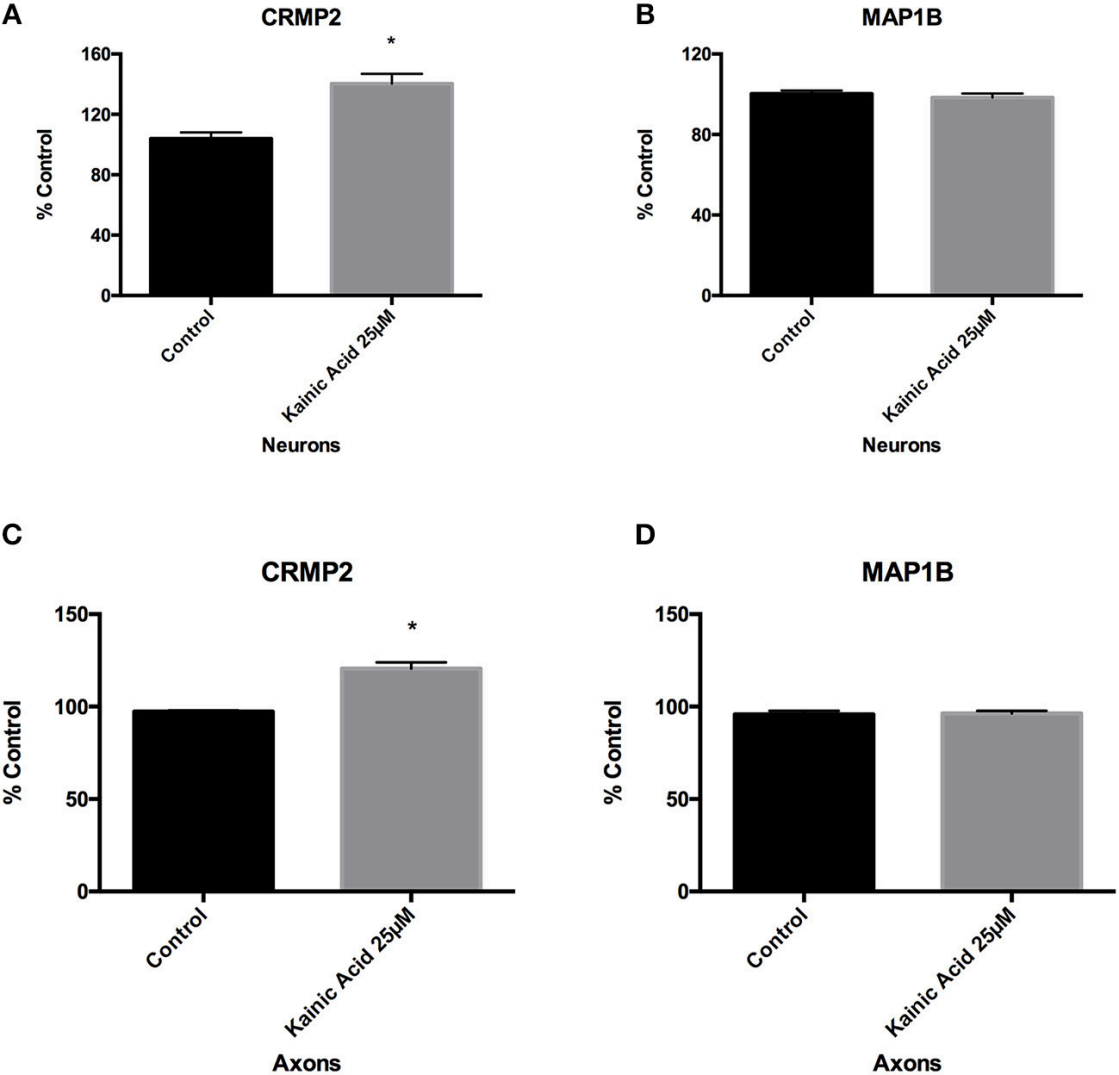
Changes to microtubules associated proteins after kainic acid treatment. Microtubule-associated proteins, CRMP2 and MAP1B levels were analyzed in neurons and axons after 25 μM kainic acid treatment for 6 h. ELISA analysis demonstrated that CRMP2 levels in **(A)** neurons and **(C)** axons were significantly increased (*p* < 0.05) relative to control. ELISA analysis of MAP1B levels in **(B)** neurons and **(D)** axons were unchanged compared to control after 25 μM kainic acid treatment. Bar graph represents mean ± SEM *p < 0.05 relative to control.

Since 25 μM kainic acid treatment affected levels of the microtubule associated protein tau, we examined whether other microtubule associated proteins were altered at 6 h post treatment. Neuronal cells grown on 12-well trays and cells grown in somatodendritic compartment of microfluidic chambers were exposed to 25 μM kainic acid for 6 h. CRMP2 and MAP1B levels in neurons and axons were examined using ELISA. Like tau, CRMP2 was significantly (*p* < 0.05) increased following excitotoxicity (Figures 3A,C) and MAP1B was unchanged in both neurons and axons (Figures 3B,D).

### Trichostatin A and microtubule alterations

Since our data showed significantly reduced acetylated tubulin levels in the axon after 25 μM kainic acid treatment (Figure 2), we hypothesized that promoting microtubule acetylation could rescue these changes and prevent axon degeneration. To promote microtubule acetylation, trichostatin A was used to inhibit the de-acetylating enzyme HDAC6.

**Figure 4:**
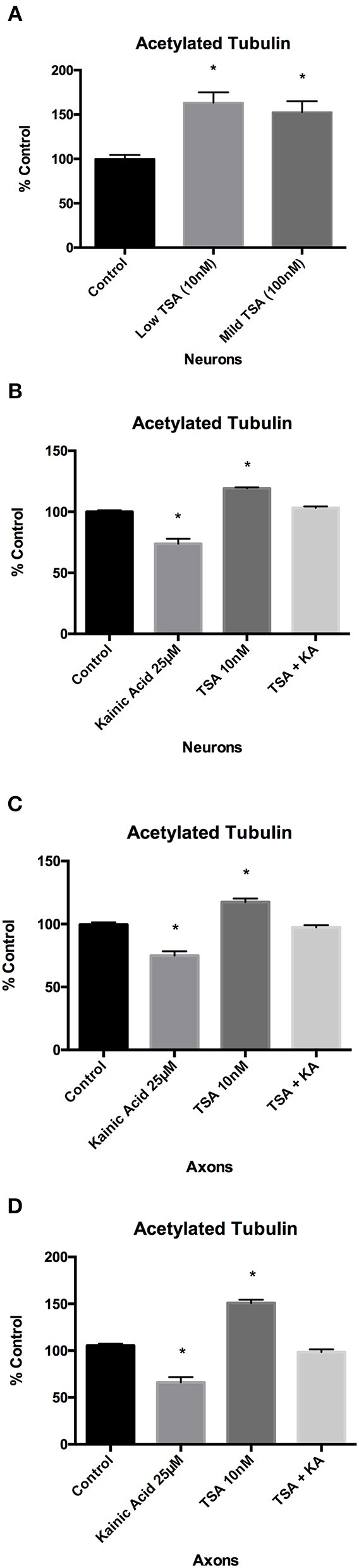
Effect of trichostatin A on microtubule acetylation following kainic acid treatment. Acetylated tubulin levels in whole cultured neurons were analyzed after 10 and 100 nM of trichostatin A treatment for 2 h. **(A)** ELISA analysis of acetylated tubulin levels after 10 or 100 nM trichostatin A demonstrated a significant increase (*p* < 0.05) compared to control. ELISA analysis demonstrated that in **(B)** whole cultured neurons and **(C)** isolated axons, 10 nM trichostatin A inhibited the effect of 25 μM kainic acid on acetylated tubulin levels, and the levels of acetylated tubulin levels in kainic acid treated neurons which had been treated with trichostatin A were not significantly different from control, despite the significant decrease (*p* < 0.05) in acetylated tubulin in cells treated with kainic acid alone. **(D)** To confirm the ELISA analysis of acetylated tubulin levels a Western blot was performed of harvested isolated axons from neurons treated with 25 μM kainic acid in the presence of absence of axonal trichostatin A (10 nM). This confirmed that trichostatin A rescued the decrease in acetylation resulting from kainic acid treatment. Bar graph represents mean ± SEM *p < 0.05 relative to control. TSA, trichostatin A; KA, kainic acid.

We first determined the concentrations of trichostatin A required to promote acetylation in our primary mouse cortical cultures. ELISA analysis demonstrated that acetylated tubulin levels after 10 and 100 nM trichostatin A were significantly (*p* < 0.05) increased compared to control at 2 h post-treatment (Figure 4A).

We next determined whether HDAC6 inhibition with trichostatin A could rescue acetylated tubulin following excitotoxin exposure. Neuronal cells grown on 12-well trays and the axonal compartment of microfluidic chambers were pre-treated with low (10 nM) trichostatin A for 2 h prior to exposure to 25 μM kainic acid. ELISA analysis demonstrated that 10 nM trichostatin A restored acetylated tubulin levels after kainic acid exposure, as compared to control, in both neurons and axons (Figures 4B,C). Western blot analysis also demonstrated that 10 nM trichostatin A restored acetylated tubulin levels after excitotoxicity in axons (Figure 4D).

**Figure 5:**
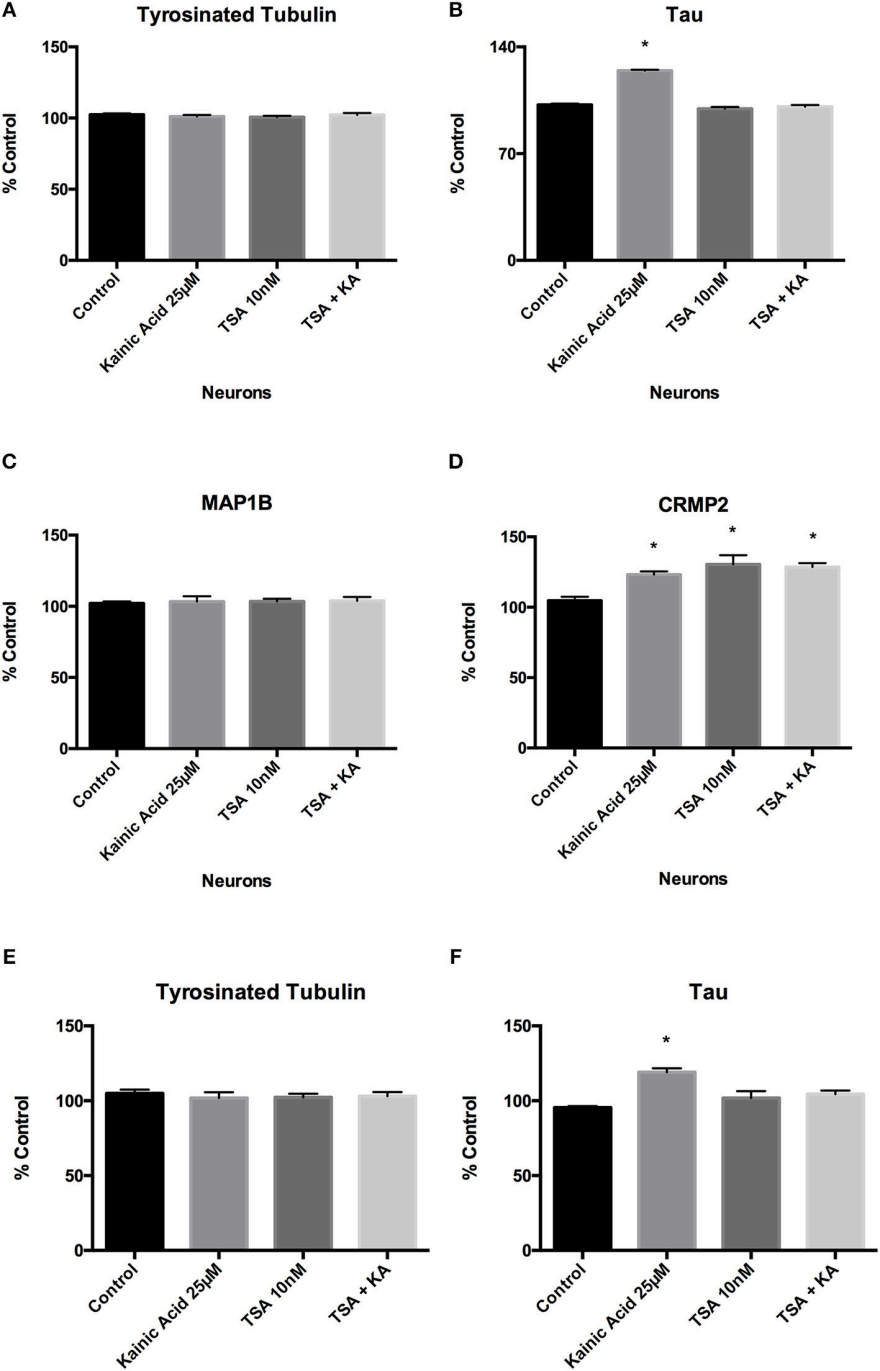
Effect of trichostatin A on tyrosinated tubulin and microtubule-associated proteins following kainic acid treatment. ELISA analysis demonstrated that **(A)** trichostatin A had no effect on neuronal tyrosinated tubulin levels following kainic acid treatment relative to control. **(B)** Trichostatin A rescued kainic acid-induced increases in tau, with no significant difference in tau levels for kainic acid treated cells in the presence of trichostatin A. **(C)** MAP1B levels were unchanged compared to control with any of the treatments. **(D)** CRMP2 levels were significantly increased (*p* < 0.05) relative to control by either treatment with kainic acid or trichostatin A or by kainic acid in the presence of trichostatin A. Tyrosinated tubulin and tau levels were also analyzed in isolated axons after treatment of the neurons with 25 μM kainic acid in the presence or absence of axonal 10 nM trichostatin A. **(E)** Tyrosinated tubulin levels were not significantly different between treatment groups. **(F)** As expected, tau levels were significantly increased (*p* < 0.05) after 25 μM kainic acid treatment, compared to control and this effect was prevented by trichostatin A treatment. Bar graph represents mean ± SEM *p < 0.05 relative to control. TSA; trichostatin A, KA; kainic acid.

Since our results had shown that tau and CRMP2 levels were increased by kainic acid, we examined the effect of trichostatin A on these MAPs. ELISA analysis of neurons demonstrated that 10 nM trichostatin A treatment restored tau to control levels in the presence of kainic acid (Figure 5B). However, CRMP2 levels remained significantly (*p* < 0.05) higher following kainic acid in the presence of 10 nM trichostatin A (Figure 5D). As we had previously shown, tyrosinated tubulin and MAP1B levels were unaffected by kainic acid and these were also unchanged at 10 nM trichostatin A treatment (Figures 5A,C). ELISA analysis of axons also demonstrated that tau levels were restored after 10 nM trichostatin A, and tyrosinated tubulin levels were unchanged (Figures 5E,F).

### Trichostatin A and axon degeneration

**Figure 6:**
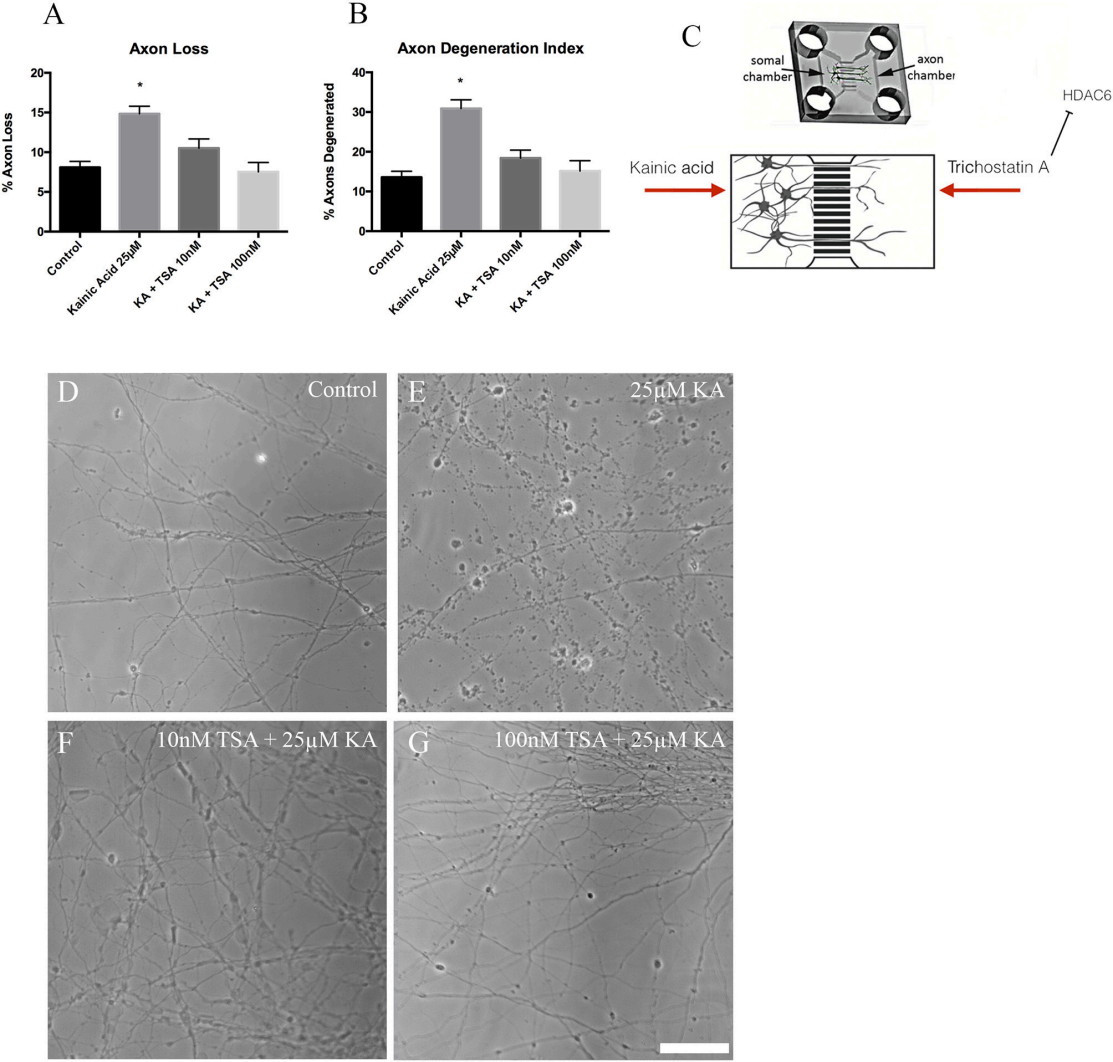
Axon degeneration and axon loss after kainic acid treatment in the presence of trichostatin A. Live images of the axonal compartments of microfluidic chambers were analyzed for **(A)** axon loss and **(B)** axon degeneration after treatment of the neurons with 25 μM kainic acid in the presence or absence of 10 or 100 nM axonal trichostatin A. Both axon loss and axon degeneration were significantly increased (*p* < 0.05) with 25 μM kainic acid treatment, however, this effect was rescued by treatment with either concentration of trichostatin A. In the presence of trichostatin A, axonal loss and degeneration were not significantly different from controls. **(C)** Figure to show how trichostatin A and kainic acid treatment is administered to microfluidic chamber compartments. Representative images for **(D)** control, **(E)** 25 μM kainic acid, **(F)** 10 nM trichostatin A + 25 μM kainic acid and **(G)** 100 nM trichostatin A + 25 μM kainic acid. Bar graph represents mean ± SEM *p < 0.05 relative to control. Scale bar = 50 μm. TSA, trichostatin A; KA, kainic acid.

We next determined if the rescue of acetylation by trichostatin A treatment following excitotoxin exposure was also protective against axonal degeneration (Figure 6). Neurons were grown in microfluidic chambers and axons exposed to low (10 nM) and high (100 nM) trichostatin A for 2 h prior to somatodendritic treatment with 25 μM kainic acid. Axon degeneration was quantitated from images acquired before and 18 h after kainic acid treatment. In the presence of 10 and 100 nM trichostatin A, axon degeneration (Figure 6B) and axon loss (Figure 6A) were significantly reduced (*p* < 0.05) after exposure to 25 μM kainic acid.

## Discussion

Understanding the mechanisms underlying the relationship between microtubule alterations and axon degeneration can potentially lead to a better understanding of neurodegenerative changes linked to excitotoxicity. The current study investigated whether excitotoxin-induced axon degeneration caused alterations in the post-translational modifications of microtubules and the expression of microtubule-associated protein, tau, and whether inhibition of these biochemical changes to these cytoskeletal proteins can protect axons.

The main finding of this study was that, following excitotoxin exposure, there were changes to both microtubule PTMs and to the levels of MAPs expressed by neurons and that these changes occurred within 6 h of treatment. The key change to PTMs was the acetylation of tubulin which occurred by 6 h and was present in both whole neuron analysis as well as within the axons. The major enzyme involved in tubulin acetylation state in mammals is alpha-tubulin acetyltransferase 1 (αTAT1; Coombes et al., 2016). It is currently unclear whether loss of tubulin acetylation in the current project results from increased deacetylation or from decreased acetylation. Furthermore, downstream processes following kainic acid exposure that result in loss of acetylation are currently not known but will be the subject of future investigations.

In the current study, treatment with trichostatin A prevented decreases in acetylated tubulin levels after kainic acid exposure, consequently reducing axonal fragmentation and axon loss, suggesting that tubulin deacetylation is a key event in excitotoxin induced axon degeneration. Trichostatin A is a HDAC6 inhibitor, which has been investigated in motor neuron degeneration (Yoo et al., 2011; Lazo-Gómez et al., 2013) and used to treat retinal degenerative diseases (reviewed in Zhang et al., 2015). The importance of acetylation in microtubule stability has been widely reported (Howes et al., 2014; Portran et al., 2017). It is known that acetylated tubulin protects microtubules against mechanical aging and is also a marker of long-lived microtubules (Portran et al., 2017). Increasing microtubule acetylation using trichostatin A, has been shown to restore axonal transport in rat cortical neurons, following exposure to mutant leucine-rich repeat kinase (LRRK2) (Godena et al., 2014). The same study showed that *in vivo*, trichostatin A treatment or knockdown of HDAC6 and Sir-related protein (SIRT) 2 (Sirt2), rescued axonal transport in a Drosophila model of mutant LRRK2. In cultured mouse cerebellar granule cells, levels of acetylation were compared between Wallerian degeneration slow (*Wld*^*S*^) mice, which have a mutation that slows Wallerian degeneration (Lunn et al., 1989) and wildtype mice (Suzuki and Koike, 2007). The authors found that base levels of acetylation were increased in *Wld*^*S*^ cells compared to wildtype. Furthermore, the authors showed that the deacetylating enzyme SIRT2 plays a key role in resistance of *Wld*^*S*^ cells to axon degeneration, whereby they found decreased SIRT2 in *Wld*^*S*^ granule cell cytoplasm. Additionally, SIRT2 knockdown enhanced microtubule acetylation and reduced axon degeneration in wildtype granule cells. These findings also highlight the importance of microtubule acetylation in axon degeneration.

Our study also showed that tyrosinated tubulin is unchanged following kainic acid exposure. In contrast to acetylation, which is associated with long-lived microtubules, tyrosination is present in highly dynamic microtubules at the proximal end of the axon (Witte et al., 2008), and has been reported as a destabilizing microtubule PTM (Kreis, 1987; Khawaja et al., 1988). This suggests that destabilizing microtubules through tyrosination is not the key factor in driving excitotoxin-induced axon degeneration. However, in axon regeneration studies, absence of tubulin tyrosine ligase, which promoted tubulin tyrosination, has been shown to severely reduce axon regeneration (Song et al., 2015), suggesting that tubulin tyrosination may be more important for regeneration rather than degeneration. However, PTMs have also been described following developmental axon pruning, which can be modeled *in vitro* by trophic factor withdrawal (reviewed in Saxena and Caroni, 2007). Developmental axon pruning has been shown to involve decreases in both acetylation and tyrosination, with an accompanying increase in detyrosinated tubulin (Unsain et al., 2018).

The microtubule-associated protein, tau, has been implicated in neurodegenerative disease, particularly Alzheimer's disease (AD) and was therefore of interest in this study. In AD, tau has been reported to dissociate from microtubules; thereby reducing microtubule stability (Sontag et al., 1996; Kadavath et al., 2015). The dissociated tau then aggregates to form tau neurofibrillary tangles; a major hallmark of Alzheimer's disease. However, the exact function of tau in binding to microtubules is not clear. In this study, we showed that tau expression levels were increased following excitotoxicity. Tau was shown to be increased as early as 1 h after kainic acid treatment, suggesting it could be a compensatory mechanism, potentially to increase microtubule stability. The changes in tau prompted us to examine expression levels of other MAPs and we demonstrated that like tau, CRMP2 was increased following excitotoxin exposure. The dysregulation and hyperphosphorylation of CRMP2 has been observed in Alzheimer's disease (Williamson et al., 2011; Hensley and Kursula, 2016). Examining phosphorylation of these MAPs as well as how these proteins are altered by excitotoxic treatment, will be of interest in future studies.

Alterations to microtubules have also been demonstrated in other forms of axonal injury. For example, dynamic stretch injury of neuronal cultured cells induced axon degeneration, which was inhibited by taxol (Tang-Schomer et al., 2010). This strengthens earlier research in this area, suggesting that cytoskeletal changes begin as early as 5 min post fluid percussive injury (Pettus and Povlishock, 1996), indicating that changes to microtubules could be one of the earliest events to occur after initiating axonal injury and degeneration. Another study which investigated alpha tubulin levels after optic nerve stretch injury found decreased alpha tubulin levels between 0.5 and 4 h post-injury, and a secondary decline after 72 h post-injury (Serbest et al., 2007). This also suggests that microtubules may have an initial role after injury and another role in a secondary event post-injury.

## Conclusion

Together these data indicate that alterations to microtubule may be an early and modifiable event in several forms of axon degeneration. The earliest change detected, after 1 h of kainic acid treatment, was a significant increase in tau, which was followed by 6 h by a significant decrease in acetylated tubulin and significant increase in CRMP2. These MAPs and PTM are associated with dynamic instability of the microtubules, however tyrosinated tubulin, and MAP1B levels, which are also linked to microtubule stability were unaffected. Together these results suggest that microtubule alterations were specific to the insult. Promotion of microtubule acetylation with trichostatin A treatment, in some cases restored altered levels of PTMs and MAPs to control levels. The current study demonstrates that altering microtubule acetylation can help prevent axon degeneration through excitotoxin exposure. Further deciphering microtubule post-translational modifications and their interaction with degenerating pathways will be important in providing therapeutic treatment in several neurodegenerative diseases.

## Author contributions

KH, JV, and AK conceived and designed the experiments; KH and NT performed the experiments; KH analyzed the data; JV and AK contributed reagents, materials, analysis tools, KH, AK, and JV wrote the paper.

### Conflict of interest statement

The authors declare that the research was conducted in the absence of any commercial or financial relationships that could be construed as a potential conflict of interest.

## Supplementary material

Supplementary Figure 1 **(A)** Western blots of axons from microfluidic chambers after treatment with 2 h trichostatin A and 6 h somal kainic acid. The Western blots were quantitated in Figure 4D. **(B)** alamarBlue cell viability assay after treatment with trichostatin A for 2 h and **(C)** after 6 h kainic acid treatment. Bar graph represents mean ± SEM ^**^*p* < 0.01; ^****^*p* < 0.001 relative to control. TSA, trichostatin A; KA, kainic acid.
