# Supplementary figures and images for: The HDAC6 Inhibitor Trichostatin A Acetylates Microtubules and Protects Axons From Excitotoxin-Induced Degeneration in a Compartmented Culture Model
Source: Front Neurosci. 2018 Nov 29;12:872. doi: 10.3389/fnins.2018.00872 (PMC6282003; doi:10.3389/fnins.2018.00872)

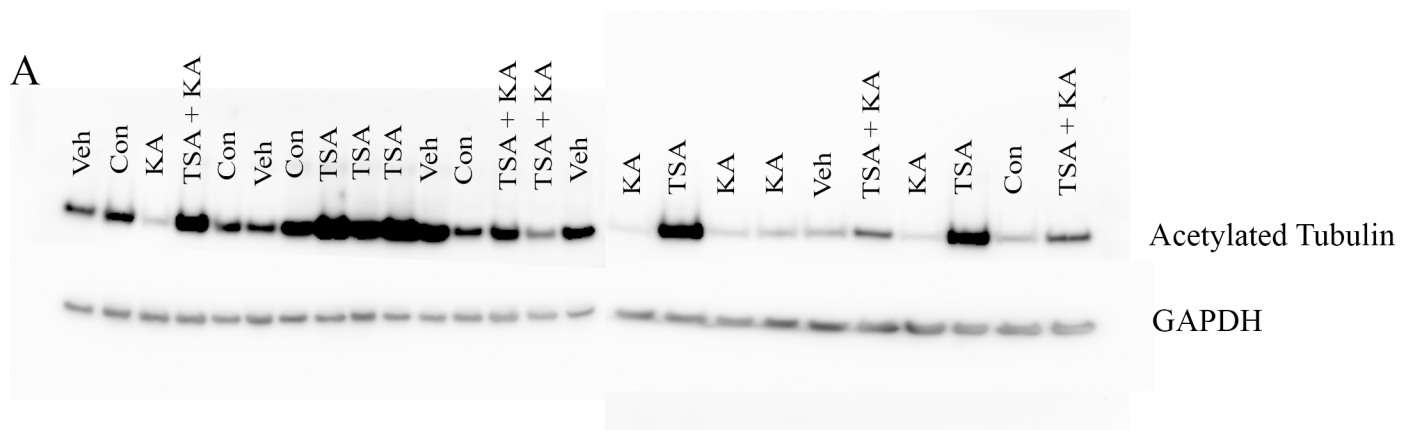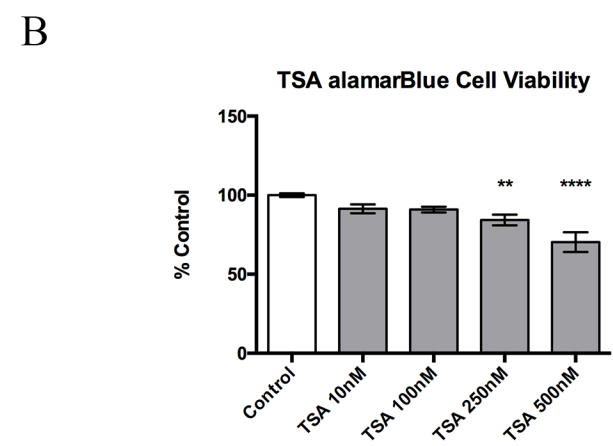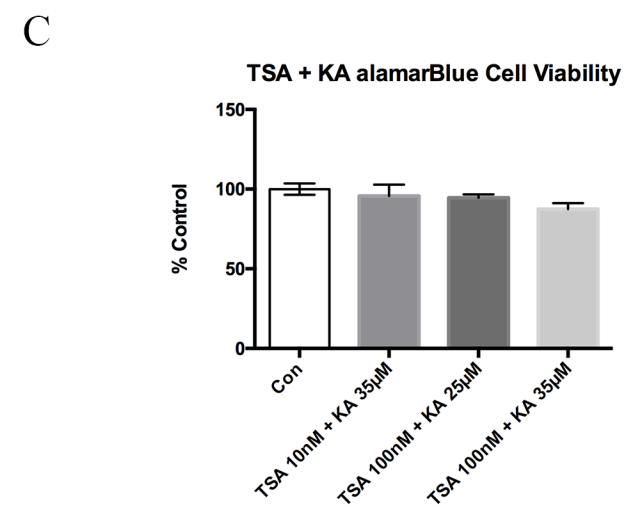

Supplement: Supplementary Figure 1 — (A) Western blots of axons from microfluidic chambers after treatment with 2 h trichostatin A and 6 h somal kainic acid. The Western blots were quantitated in Figure 4D. (B) alamarBlue cell viability assay after treatment with trichostatin A for 2 h and (C) after 6 h kainic acid treatment. Bar graph represents mean ± SEM **p < 0.01; ****p < 0.001 relative to control. TSA, trichostatin A; KA, kainic acid. [file Data_Sheet_1.PDF]
